# What Encourages Longer Educational Careers in Tertiary Education? A Three-Level Approach for the Case of Romanian Universities

**DOI:** 10.3390/ijerph182312864

**Published:** 2021-12-06

**Authors:** Ana-Maria Zamfir, Cristina Mocanu, Adriana AnaMaria Davidescu

**Affiliations:** 1 Department of Education, Training and Labour Market, National Scientific Research Institute for Labour and Social Protection, 010643 Bucharest, Romania; anazamfir2002@yahoo.com (A.-M.Z.); mocanu@incsmps.ro (C.M.); 2 Department of Statistics and Econometrics, Bucharest University of Economic Studies, 010374 Bucharest, Romania

**Keywords:** tertiary education, educational careers, three-level approach, logistic regression model, Romanian universities, educational survey

## Abstract

Students’ commitment and engagement in the educational process are shaped by a dense combination of factors, with effects on educational attainment and on the length of their educational careers. Decisions of prolonging education by enrolling in master’s degrees are beneficial for both individuals and societies, as such programs provide higher levels of specialized skills Longer educational careers are favored by a mix of factors acting at the level of individual, university, or wider environment. We focus our study on exploring factors conducive for students’ intentions to pursue master’s degrees considering longer educational careers as desirable outcomes. Thus, this article investigates how the individual and environmental factors interplay and shape the predisposition of students to prolong their educational career by enrolling in master’s degrees. For this, we applied three-level logistic regression models for a sample of 502 students enrolled in their final year of bachelor studies grouped by universities and universities grouped by counties. The empirical results revealed that the final grade, the father level of education, the type of working contract, and job seniority are individual-level determinants influencing the decision of enrolment in a master’s program. At the university level, the type of university and the university performance score positively impact the students’ decision to enroll in a master’s program. At the county level, the empirical evidence pointed out the significance of determinants such as the proportion of students enrolled in bachelor studies; participation rate in education and training; employment level in high-technology sectors (HTC), total-knowledge intensive sectors (KIS), and knowledge-intensive high-technology sectors (KIS_HTC); proportion of persons with tertiary education employed in science and technology; proportion of scientists and engineers; local development; R&D expenditure, personnel, and researchers in the business sector; average gross earnings; density of active firms; birth rate of companies; proportion of innovative enterprises or those introducing product innovations on the decision to enroll in a master’s program.

## 1. Introduction

We are part of a social system in which change fully characterizes each component part, including practically every action and approach undertaken within the social groups of affiliation, the individual human being thus both part of the change and its promoter. For change to respond to the needs and desires of social progress, it is mandatory to embed the right mix of skills [1].

Skills development is a lifelong process through which we acquire and upgrade our skills, competencies, and qualifications (relative to change), in line with the emerging needs of the labor market and society [2]. People need to possess key competences and basic skills (languages and digital skills), as well as relevant specific skills, as the needs of the labor market evolve. More recently, transversal skills (how to learn and take initiative, to work with others, and problem solving) became increasingly important for developing a successful career. Digital technology is transforming almost every aspect of our public, private, or professional lives. For every individual employee, student, and citizen, this technological innovation creates a demand for new and evolving digital skills. Ensuring the skills needed for the workforce is a key component in meeting the three priorities of the Europe 2020 Strategy.

Educational systems need to provide the necessary skills, which include specialized competences, digital and transversal skills, media education, and communication in a foreign language. Harnessing the talents and potential of future employees is very important and requires better cooperation between the labor market and the education system, but also greater transparency in the labor market beyond traditional approaches that measure skills only through formal qualifications [1]. Enrolling in a graduate education program allows students to expand their knowledge in a specific field of study while also developing in ways to prepare them for their future career [3]. Sometimes, recently enlisting undergraduate students must choose which graduate school they ought to go to. Due to the complex nature of selecting a graduate program, undergraduate students must make their choice through reflection and recognizable proof of individual, scholarly, and career objectives [4].

The main aim of the article refers to educational decisions, with a focus on the transition from bachelor to master studies, exploring the individual, institutional, and local factors that shape the predisposition of bachelor students when enrolling in master’s programs, considering that this decision is influenced by a dense combination of mechanisms.

In this context, our study is focused on the following research questions:

Q1: What is the role of individual factors (academic performances, familial circumstances, gender, age, area of residence, participation in labor market, and expected employment and earnings) in shaping students’ intentions to pursue master’s degrees?
Q2: Do university-level factors influence the propensity of students in enrolling in master’s programs?
Q3: How does the wider economic and social environment determine the predispositions of students to pursue master’s degrees?
Q4: Do the individual, university, and socio-economic factors interact in shaping students’ intentions to pursue master’s degrees?
Q5: Does the decision of enrolment to a master’s program vary across universities? Across counties?

The choice to seek post-graduation instruction is a decision made by thousands of people after completion of their undergraduate degree [2]. When graduate students effectively complete their graduate degrees, they encounter an increment in profit and quality of life [5]. While the research field of graduate education and its determinants has grown in size and importance, few studies have focused on the factors shaping the enrolment in master’s degrees. Moreover, not many studies on educational decisions have been carried out in Central and Eastern European and Balkan countries. The current paper is valuable for filling a gap in the current research, as no study has been carried out on identifying factors influencing the decision of enrolment to a master’s program in Romanian universities. Romania represents an interesting case as a country with an emerging economy and expansion of education, including a raising participation in higher education.

The paper is structured as follows. The section of the literature review offers an overview of the most relevant studies concerning the drivers of the transition from bachelor to master studies, while Section 3 offers additional information about the data used in the analysis, providing also a brief description of the methodology used within the paper. Section 4 is dedicated to the presentation of the main results, highlighting also a sub-section of discussions in relation to the main findings. The paper ends with concluding remarks and highlights both limitations and recommendations for further study.

## 2. Literature Review and Theoretical Framework

“Higher education is considered as incubator of the technical progress as well as the provider of higher-level skills” ([6], p. 385). Thus, the topic of this article is related to the study of educational decisions, with a focus on the transition from bachelor to master studies. While earlier studies focused on the concept of student retention into higher education, recent theoretical frameworks rely on the idea that the choice of enrolment into master’s degree is a new and distinct decision [2]. Educational decisions are shaped by a combination of mechanisms covering individual preferences and opportunities, together with the conditions that influence them. Hosler and Gallagher [7] developed the three-phase college-going model in which the educational choice process includes a first stage called the predisposition phase. In this phase, students decide whether or not they want to continue their education by enrolling in a higher-level education program. This paper explores individual, institutional, and local factors that shape the predisposition of bachelor students to enroll in master’s programs, considering that this decision is influenced by a dense combination of mechanisms. We develop our analysis in the framework of the cultural and social capital role, as well as of the human capital theory.

Considering the relevance of cultural and social capital for educational choices, previous studies found that various individual factors influence such decisions. One important factor is related to the previous academic performances of students. They influence expectations with respect to future academic success and perceived academic self-efficacy, and determine the predisposition of students to take risks related to enrolment in a higher-level educational program [8,9]. Familial circumstances represent another group of factors shaping educational careers. On the one hand, students with higher family income display a higher probability to attain higher levels of education [10,11]. On the other hand, educational attainment of the students’ parents represents a measure of their cultural capital that influence the probability of students to graduate in higher levels of education. Those having parents with higher levels of education register higher educational attainment [9,12]. Previous findings show that the education of parents also indirectly influence the enrolment in master’s programs through characteristics of educational institutions, academic performances, and academic expectations [9]. The cultural model for the parents of the cohort covered in the survey was extensively a conservative one, characterized by higher ages and levels of education for the male partners within the married couples, as well as by low levels of births outside marriage. As our study addresses the decision of youth to extend their educational pathway, we considered the father’s level of education as a better proxy for the stock of education existing within households.

Additionally, some socio-demographic characteristics such as gender, age, and race, as well as their residence area influence students’ decisions to enroll in higher levels of education, including master’s degrees [10,13,14].

Another category of factors that influence educational careers are related to the characteristics of educational institutions [10]. According to the theory of students’ retention [15], students experiencing a match between their motivations and abilities, and the academic and social qualities of educational institutions influence in a positive manner students’ academic and social integration. Previous studies found that the quality of the academic environment influences students’ educational decisions [16]. Another characteristic of influence is the type of university. Students from research universities have a higher propensity for pursuing master’s degrees than those enrolled in teaching-oriented universities [14]. Of course, the satisfaction of students with educational experience shapes their predisposition to continue education, while satisfaction is influenced by perceived performances and outcomes of educational institutions [17].

Another strand of factors shaping educational decisions are related to the human capital theory. According to this theory, education represents an investment in human capital that is driven by the expected economic returns [18,19,20]. Thus, education is seen as an investment that allows for the accumulation of knowledge and skills that provide people with better career prospects and access to higher earnings. Previous studies found that the demand for higher education is shaped by the unemployment level [12,21]. Additionally, educational decisions are influenced by expected earnings upon completion of a specific level of education [22,23], while situations of overeducation affect many higher education graduates [24]. Additionally, the major of students has been found to influence their enrolment in master’s programs due to differences in the expected advantages [2]. Both expected employment prospects and the income associated with graduation are taken into consideration in educational decisions [12]. Higher expected earnings are conducive for enrolment in post-graduate education [22]. In addition, participation of students in the labor market influences their decision of enrolment in higher education levels [25] as they face a tradeoff between current potential earnings and prospective higher incomes expected after graduation.

The mix and interaction of individual and macro-level factors shaping educational decisions inspired scholars to develop multilevel models for studying this topic. In analyzing the determinants of university enrolment in Vietnam, Vu et al. [6] emphasized the relevance of household socioeconomic status, gender, ethnic group, migrant status, and urban/rural residence as individual-level predictors of participation, as well as the relevance of fertility stabilization, income distribution, and average education level as contextual predictors at the provincial level.

Beyene and Yimam [26] investigated the drivers of academic achievement in higher education universities by applying a multilevel approach, emphasizing that the university entrance exam result, sex, mother’s education level, father’s education level, drug use, number of assessments, and group study status are the most relevant determinants of academic achievement.

Using a qualitative methodology and two focus groups, Sturm ([27], p.122) identified the factors that most influence enrolment in graduate education: the “need of the graduate degree for a desired career, desire to gain qualifications that would result in more opportunities, the institution being able to meet financial concerns, and the personal touch/comfort they felt from the institution”.

Students’ commitment and engagement in the educational process are shaped by a dense combination of factors, with effects on educational attainment and the length of their educational careers. Many studies on educational decisions employ binomial logit models considering that students choose the most attractive option from two alternative choices [12,28]. Taking into account the multi-layers of factors shaping educational decisions, more recent studies apply multilevel models for studying the complex mechanisms that shape educational decisions, especially in relation to higher education [2]. Such multilevel models include different layers of factors from individual, education institution, and local labor market levels [2].

## 3. Methodology and Data

In order to capture the intention of enrollment to a master’s program in the academic year of 2020/2021, data were gathered from an educational survey. Data collection took place in 2019 via a questionnaire-based survey among 502 students enrolled in their final year of bachelor studies. The survey covered students enrolled in engineering and social sciences in ten Romanian universities from five countries. Students have been asked to report their intentions of further pursuing a master’s level degree in the school year of 2020/2021. The survey also collected a variety of data on students’ characteristics such as gender, age, area of residence, academic performance, parents’ education, and relation to the labor market, as well as a set of subjective information regarding their expectations and perceptions on the monetary and non-monetary benefits brought by a master’s degree. Additional information on the characteristics of educational institutions providing bachelor studies (performances of the university and supply of master’s programs) have been retrieved from administrative sources.

For investigating other potential factors that could encourage longer educational careers in tertiary education, we structured a set of variables on three main pillars, namely labor market, higher education, and innovation, in order to capture how the specificities of the context could influence such a decision to continue the educational pathway. As the study is focused on the decision to pursue master’s programs, we extended the list of variables characterizing the labor market in order to capture the demand for highly specialized skills (Table 1). Taking into account the fact that the counties selected in the analysis were representative for the regions and the fact that county-level variables were rather limited, we decided to complement our analysis, including also regional-level indicators. Thus, the following were included: the ILO youth unemployment rate, vacancy rates, employment rate for ISCED 5–8, employment in high-technology sectors, employment in total knowledge-intensive services, employment in knowledge-intensive high-technology services, persons with tertiary education (ISCED) and those employed in science and technology, proportion of scientists and engineers, participation rate in education and training, intramural R&D expenditure (GERD) in the higher education sector, intramural R&D expenditure (GERD), the business enterprise sector, intramural R&D expenditure (GERD), the public sector, total R&D personnel and researchers in the business sector, innovative firms, enterprises introducing product innovations, process innovations, and enterprises introducing product and process innovations. A detailed description of all the variables within the model is provided in Table 1.

### Multilevel Econometric Modelling

In order to identify the main determinants of Romanian students’ intention of applying to a master’s program, three-level logistic regression analysis based on the hierarchical structure of the data, with individuals (level 1) grouped by universities (level 2) and universities grouped by counties (level 3), was applied. The command in STATA 15 for the estimation of multilevel logistic regression models is xtmelogit. This type of analysis uses individual-level variables and explores whether each university size-level independent variable together with county level indicators is significantly associated with the intention of applying to a master’s program.

Thus, by using a three-layer multilevel model, we captured the relative importance of universities and labor market variables as influences on the decision to apply to a master’s program, paying particular attention to the assessment of potential causal effects of universities performances and local labor market opportunities. The main hypothesis behind the model is that individual, university, and socio-economic factors interact in shaping students’ intentions to pursue master’s degrees.

**Figure 1:**
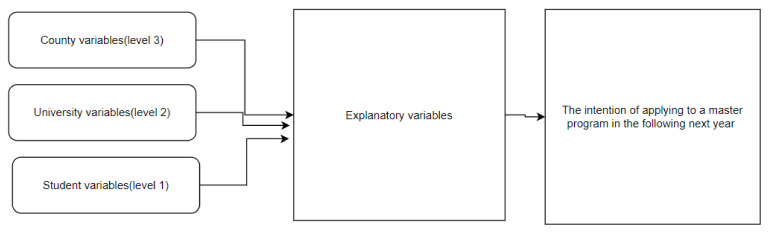
Conceptual framework of the main determinants for the intention of applying to a master’s program.

The conceptual framework of the main determinants for the intention of applying to a master’s program is presented in Figure 1.

The methodology of building the three-level models followed a stepwise approach. The first stage implied the estimation of a baseline random intercept model with no explanatory variables in order to identify whether a multilevel approach was appropriate. This model included only an intercept as well as university and county random effects, and an individual-level residual error term; the model makes no adjustments for predictor variables.

(1) $$\log\left(\frac{\pi_{ijk}}{1-\pi_{ijk}}\right)=\beta_{0}+v_{k}+u_{jk}+e_{ijk}$$

$v_{k}\sim N\left(0,\;\sigma_{v}^{2}\right)$
$u_{jk}\sim N\left(0,\;\sigma_{u}^{2}\right)$
$e_{ijk}\sim N\left(0,\sigma_{e}^{2}\right)$

where the log odds are the logarithm of the odds (i.e., the ratio between a probability value (Phi) and its complementary) for individual *i*(*i* = 1,…,476) in university *j*(*j* = 1, …10) and in county *k*(*k* = 1,…,5); $\beta_{0}$ is the mean across all counties; 𝑣𝑘 is the effect of county 𝑘; 𝑢𝑗𝑘 is the effect of university 𝑗; and 𝑒𝑖𝑗𝑘 is the individual-level residual error term. The county, university effects, and the individual level residual errors are assumed independent and normally distributed with zero means and constant variances.

In interpreting variance components in multilevel models, we considered the variance partition coefficients (VPCs), which report the proportion of the observed response variation that lies at each level of the model hierarchy (Leckie, [29]). They therefore allowed us to establish the relative importance of universities, counties, and students as sources of variation of students’ decision to enroll in a master’s program.

The second stage involved constructing a model at first-level (i.e., individual-level and university) variables in an attempt to understand their effects and thus to test the impact of individual characteristics:

(2) $$\log\left(\frac{\pi_{ijk}}{1-\pi_{ijk}}\right)=\beta_{0}+\beta_{1}\cdot X_{ijk}+\beta_{2}\cdot X^{\prime }{}_{jk}+v_{k}+u_{jk}+e_{ijk}{}_{}$$

$v_{k}\sim N\left(0,\;\sigma_{v}^{2}\right)$
$u_{jk}\sim N\left(0,\;\sigma_{u}^{2}\right)$
$e_{ijk}\sim N\left(0,\sigma_{e}^{2}\right)$

In the third step, the logit random intercept model specification, including individual and university-level explanatory variables, as well as county-level explanatory variables, is the following:

(3) $$\log\left(\frac{\pi_{ijk}}{1-\pi_{ijk}}\right)=\beta_{0}+\beta_{1}\cdot X_{ijk}+\beta_{2}\cdot X^{\prime }{}_{jk}+\beta_{3}\cdot X^{\prime \prime }{}_{k}+v_{k}+u_{jk}+e_{ijk}{}_{}$$

$v_{k}\sim N\left(0,\;\sigma_{v}^{2}\right)$
$u_{jk}\sim N\left(0,\;\sigma_{u}^{2}\right)$
$e_{ijk}\sim N\left(0,\sigma_{e}^{2}\right)$

As in any regression model, we can include interaction effects which allow for the possibility that the effect of one explanatory variable on the outcome can depend on the value of another explanatory variable. Furthermore, it was worth to test if the effect of contextual county factors on the decision of applying to a master’s program depended on the type of university (engineering vs. social). Therefore, in the fourth step, different random intercept models with cross-level interactions were estimated.

(4) $$\log\left(\frac{\pi_{ijk}}{1-\pi_{ijk}}\right)=\beta_{0}+\beta_{1}\cdot X_{ijk}+\beta_{2}\cdot {X^{\prime }}_{jk}+\beta_{3}\cdot X^{\prime \prime }{}_{k}+\beta_{4}\cdot X^{\prime }{}_{jk}\cdot X^{\prime \prime }{}_{k}+v_{k}+u_{jk}+e_{ijk}{}_{}$$

$v_{k}\sim N\left(0,\;\sigma_{v}^{2}\right)$
$u_{jk}\sim N\left(0,\;\sigma_{u}^{2}\right)$
$e_{ijk}\sim N\left(0,\sigma_{e}^{2}\right)$

**Figure 2:**
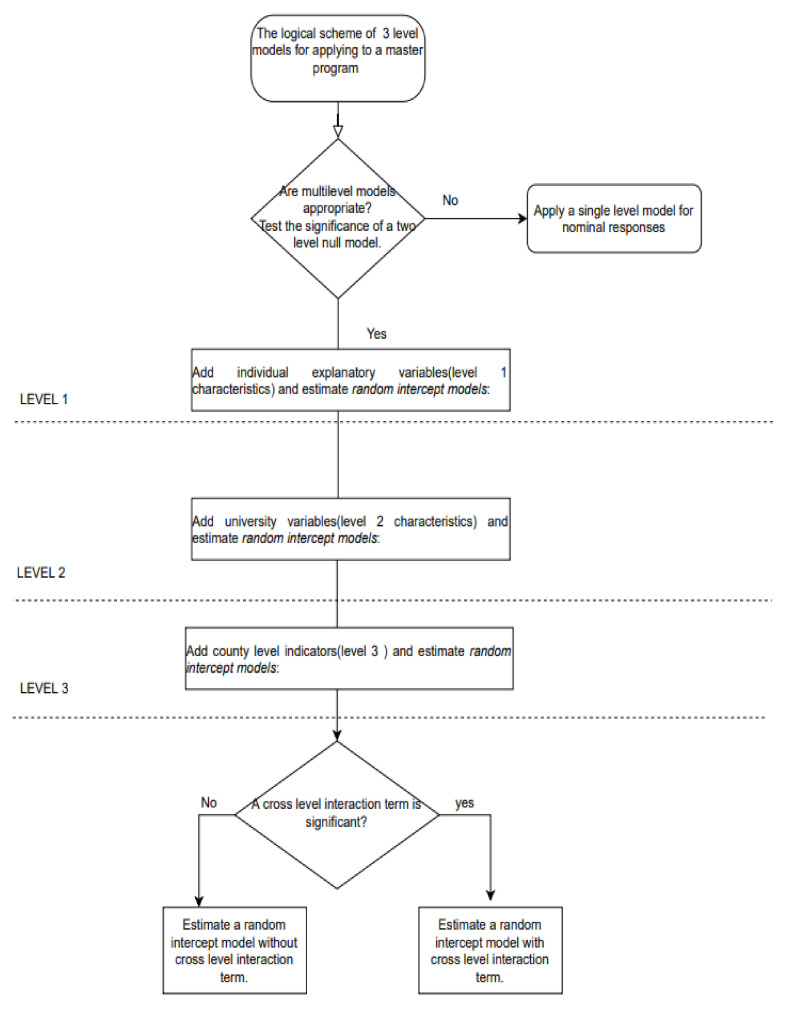
The general scheme of three-level models for applying to a master’s program.

The general scheme of three-level models for applying to a master’s program is displayed in Figure 2. Different specifications of three-level logistic models were estimated in order to overcome the issue of multi-collinearity, as some of the university-level and county-level variables could be correlated. The software used for the estimation was STATA version 15 [30].

## 4. Results

### 4.1. Descriptive Results

From the total of 502 respondents, the distribution of students among five counties was balanced, while the gender distribution revealed that almost 54.38% were women, living in urban area, and with the average age of 21.8 years old. Regarding the highest education level of the father, the majority of the respondents declared to be at a medium level (67.13%; Table 2).

The average grade among the interviewed students was 8.81, with only 25% of the students having finished their studies with a grade higher than 9.4. There are significant differences regarding the final grade among the universities included in the sample, wherein the highest final grade was registered for the University of Agronomic Sciences and Veterinary Medicine, while at the opposite end was Transylvania University. Statistically significant discrepancies were highlighted also at the county level, with Suceava being the county with the highest final grade, while Brasov occupied the last place.

As expected, most of the students declared not having significant work experience, with only 20% reporting at most two years of employment experience. At the level of the whole sample, only 15% of the respondents declared to have a full-time working contract and 22% declared to work on the basis of a part-time contract.

Data related to the subjective income show that 63% of the respondents declared that they manage their current expenses rather easily, referring to the total monthly income of the household.

In analyzing respondents’ perceptions of the potential benefits of completing a master’s program, most of them considered that the difference in terms of salary between a bachelor’s degree and master’s degree is between RON 500 and RON 1000, with statistically significant differences among counties (RON 780 in Suceava and only RON 430 in Cluj). Therefore, the average expected salary for a person with a master’s degree was RON 3815, with significant differences among counties (RON 4020 for Cluj and RON 3600 in Bucharest).

In analyzing respondents’ perceptions related to the number of unemployed individuals with a bachelor’s degree vs. master’s degree, the majority of them considered that having an additional diploma does not decrease the probability of being unemployed. For 43% of them, the difference in terms of the unemployment proportion with a master’s degree vs. bachelor’s degree is zero, while only 19% of them agreed that an additional diploma could decrease the number of unemployed individuals only by 5%. Therefore, the average proportion of unemployed persons with a master’s degree was 10%, with statistically significant discrepancies among counties (13.35% in Cluj and 7.36% in Suceava).

From the university side perspective, the study equally considered engineering and social universities, with different educational performances. Therefore, the highest institutional score was registered by Babes-Bolyai University, followed by the University of Bucharest and Politehnica University, while in terms of students enrolled in master’s programs and the number of master’s programs, at first place are the University of Bucharest, Babes-Bolyai University, and Politehnica University.

Additionally, at the county level, there were significant discrepancies, with Bucharest and Cluj being the counties with the highest level of institutional performance in terms of university score, while in terms of the number of students enrolled in master’s programs and the number of master’s programs, Cluj and Bucharest occupied the first place, with Suceava at the opposite end.

Among the students from different universities, the overall decision of applying for a master’s program in the next academic year was favorable, as 53.6% of the respondents declared that they have the intention to continue their educational career following the courses of a master’s program in the next year. The results of the Kruskal–Wallis test revealed that there was a highly statistically significant difference in the decision of applying to a master’s program among students from different universities, with a higher associated probability for those one from Babes-Bolyai University.

In analyzing the decision of applying for a master’s program in different universities and counties, the empirical results of the Kruskal–Wallis test emphasized statistically significant differences, with Bucharest being the county with the highest proportion of students wishing to continue their specialization following a master’s program, while at the opposite side was Cluj with the lowest proportion of students. Significant differences in the enrolment intentions between students from different universities and counties support the development of a three-level model incorporating individual factors as well as university and county-level characteristics.

### 4.2. The Main Results of the Estimated Models

A three-level model was used to allow for a correlation between the intention of individuals living in the same country to apply for a master’s program and the intention to explore the extent of between-county variations in master’s program enrollment intentions with students nested in universities and universities nested in counties.

The first stage required the estimation of a baseline random intercept model, with no explanatory variables, used for proofing whether this multilevel technique is adequate. This stage revealed that the log-odds of enrollment in a master’s program for an ‘average’ university from an ‘average ‘county is estimated to be $\beta_{0}=0.293$. The between-county variance of the log-odds of enrollment in a master’s program is estimated to be 0.23, while the between-university variance of the log-odds of enrollment in a master’s program is estimated as 0.809.

The LR test has been used to compare the current model to a single-level model with no county effects and no university effects. The high value of the test (Chi-square test = 33.87 with a *p*-value = 0.000) revealed that there is a significant variation between universities and counties in the proportion of those applying for a master’s program. The three-level model therefore offers a significantly better fit to the data than the single-level model.

Taking into account the between-university variance of 0.809, the variance partition coefficient (VPC) is 19.73% (the VPC is calculated as 0.809/(0.809 + 3.29) = 0.1973, thus almost 20% of the residual variation in the propensity of enrollment in a master’s program is attributable to unobserved university characteristics), indicating that almost 20% of the variance in applying to a master’s program can be attributed to differences between universities rather than counties.

Furthermore, the variance partition coefficient (VPC) based on the between-county variance (0.23) is 6.53% (as 0.823/(0.23 + 3.29) = 0.1973, with almost 7% of the residual variation in the propensity of enrollment in a master’s program is attributable to unobserved county characteristics).

After testing and revealing that the multilevel mixed-effects models are the appropriate ones, the second step is to add student-level predictor variables in Model I as well as university-level predictor variables in Models II, III, and IV, in such a way as for first-level and second-level variables, in order to capture the effects of these levels on the intention of following a master’s program, while Models V–XVIII displayed also county-level predictors together with the cross-level interaction terms.

Table 3 reports the results of the random intercept models for all models. The empirical results for the *individual-level variables* pointed out that variables such as graduation final grade, the level of education of the father, seniority, or the type of working contract significantly influenced the decision of applying to a master’s program in the near future. Thus, those who have finished their bachelor studies with a high final grade are significantly more inclined to apply for a master’s degree, while the highest level of education of the father contributed also to the decision of applying to a master’s program, which is a similar educational route to the father, creating the same decision also for the student most likely because the student has been inspired by their parent’s academic achievement and academic feedback in their academic journey [26].

In all models, seniority decreased the probability of enrollment in a master’s program, as usually students who started working during college are significantly less inclined to follow the courses of a master’s program. This is not seen, in particular, for those working with a full-time contract compared to those ones not working, as individuals working full-time are significantly more inclined to apply for a master’s program compared to those ones who work based on a part-time contract.

However, we have proved a lack of statistical significance for the association between gender, age, subjective income, residence area, the perceptions related to the full-time salary of a person who graduated with a master’s degree diploma, and the share of unemployed with a master’s degree.

In order to respond to the main research questions and keeping in mind that the *university-level variables* taken into account are correlated, three alternative models (Models II–IV) were estimated, offering additional information about how university-level indicators (performance score, type of university, number of students in the master’s programs, and the number of master’s programs) can influence the decision of the enrollment of a certain student in a master’s program in the near future. The empirical results highlighted that the decision of Romanian students to enroll in a master’s program depends on the university performance score and university type, as key university-level variables, and not on the number of students in the master’s programs or the number of programs. Therefore, students are more inclined to choose the university based on the overall meta ranking, with students from engineering universities being more inclined to enroll in a master’s program.

In order to provide more detailed perspectives on how the decision of enrollment in a master’s program differs, taking into account both the differences between universities and between counties, and considering that county-level indicators are correlated, Table 3 reports the results of the random intercept models that include the individual-level and university-level variables for Models I–IV and also county-level predictors for Models V–VXIII, together with the cross-level interaction terms.

The empirical results clearly proved that the intention of applying to a master’s program does not differ according to gender, age, residence area, subjective income, perceptions related to the full-time salary of a person with a master’s diploma, the proportion of unemployed persons with a master’s diploma, the number of students in the master’s programs, or the number of master’s programs.

In order to respond to Q1, at the individual level, this decision is more likely influenced by the final grade, high level of education of the father, and work seniority together with the full-time working contract, and the significance of these variables were preserved in all models.

Therefore, students with high average final grades are more inclined to continue their education by applying for a master’s degree and the high level of education of the father is associated with a higher probability of enrolment. If the father has a high level of education, this will increase the probability of continuing the educational career of the child.

If the work seniority acts by significantly decreasing the probability of enrolment into a master’s program, as individuals already accumulating experience in the workplace are no longer inclined to apply for a master’s degree, things differ in terms of the employment contract type. More specifically, it can be highlighted that those students working with a full-time contract tend to be more inclined to apply for a master’s program compared to those who do not work.

It is worth mentioning that *individual-level variables* such as subjective income, the full-time salary of a person who graduated with a master’s degree diploma, and the share of unemployed with a master’s degree do not exhibit any statistical influence on the decision to enroll in a master’s program considering the lack of significance preserved in all the models.

With respect to *university-level indicators and in order to respond to Q2*, the empirical results highlighted the statistical significance of all the estimated models of university type and university score on the intention of continuing the educational route. Furthermore, students from a social university are less inclined to apply to a master’s program compared to those from an engineering university. The university score positively impacts the intention of applying to a master’s program, with students being interested in selecting a higher performing university.

Finally, in order to assess the main determinants from the county level on the intention of applying to a master’s program together with the cross-level interaction term, thirteen alternative models (Models V–XVIII) are presented in the Table 3. It is worth mentioning that a significantly higher number of specifications were tested, with some of them suffering from a lack of statistical significance; only those with statistical significance are displayed in Table 3 and Table 4.

At the county level, the empirical evidence offers valuable information for research question Q3, proving the statistical significance of three pillars of the determinants: education-specific determinants; labor market-specific determinants; and innovation-related indicators.

Thus, among the educational indicators proving to empirically influence the decision of enrolment in a master’s program, we can mention the proportion of students enrolled in bachelor studies and the participation rate on education and training. Thus, an increased proportion of students enrolled in bachelor studies decreases the probability of enrolment in future master’s program because, in the process of expanding education, the perception is that the educational premium is no longer as high and they no longer find the motivation to continue their studies. However, in the case of engineering university students, an increase in the proportion of enrolled students in bachelor studies increases the probability of applying to a master’s program in the near future.

The participation rate on education and training manifested a positive and statistically significant impact on the intention of enrolment in a master’s program.

Among labor market indicators, the empirical results proved a positive and statistically significant impact on the decision of applying to a master’s program regarding a higher level of employment in high-technology sectors (HTC) or total-knowledge intensive sectors (KIS), or both (knowledge-intensive high-technology sectors (KIS_HTC)) as well as a higher proportion of persons with tertiary education employed in science and technology; a higher proportion of scientists and engineers; a higher level of local development; and a higher R&D expenditure, number of personnel, and number of researchers in the business sector.

Therefore, all these county-level determinants positively influencing the decision of continuing the educational career of a student represents a specificity of the labor market, requiring specialized employees (high-skilled employees) and capturing the formal creation of new knowledge within firms, most likely in science-based sectors where most new knowledge is created in or near R&D laboratories.

In addition, factors such as the average gross earnings, the density of active firms together with the birth rate of companies, the proportion of innovative enterprises, as well as those ones introducing product innovations tend to decrease the probability of continuing the educational career and attract students to the labor market. Innovative enterprises are enterprises launching new or significantly improved products (good or service) on the market or enterprises which introduced new or significantly improved processes, new organizational methods, or marketing methods. A product innovation is the market introduction of a new or significantly improved good or service with respect to its capabilities, user friendliness, components, or sub-systems.

Thus, when the birth rate of enterprises and density of active firms is relatively high, as well as in the case of a high average gross earnings, students will be less inclined to continue their educational pattern.

Another important result refers to the fact that the share of innovative enterprises and the share of enterprises introducing product innovations, representing a proxy for the innovative labor market and job creation, negatively impact the decision of applying to a master’s program.

Until now, the models assumed that the contextual effect of different determinants is the same for all students regardless of the university type (engineering vs. social). At this stage, we have modified this assumption, allowing for the effect of the student proportion enrolled in bachelor studies and participation rate on the education and training, regarding the decision of applying to a master’s program, to depend on the university type, including an interaction between university type and student proportion as cross-level interactions or the participation rate on education and training in the model.

The empirical results of Model V revealed that the marginal effect of the student proportion enrolled on bachelor studies on the decision to apply for a master’s program positively and significantly depends on the university type. Thus, the student proportion enrolled in bachelor studies positively influenced the decision of applying for a master’s program more effectively for students from engineering universities compared to those from social sciences universities.

The effect of student participation in education and training on the decision of applying to a master’s program depends on the university type in Model VI; the empirical results revealed that the marginal effect of lifelong learning on the decision to apply for a master’s program negatively and significantly depends on the university type. Thus, an increase in the student participation rate of education and training negatively influenced the decision of applying for a master’s program more effectively for students from engineering universities compared to those from social sciences universities.

The empirical results of most of the models pointed out the statistical significance of the contextual effect of average gross earnings on the decision to apply for a master’s program more effectively for students from engineering universities compared to those from social sciences universities. Therefore, students from engineering universities with high earnings are more inclined to enroll in a master’s program.

Therefore, in line with question Q4, we can reveal that individual, university, and socio-economic factors interact in shaping students’ intentions to pursue master’s degrees. Additionally, the empirical results provided enough evidence based on VPC values to claim that the decision of enrolment to a master’s program varies across both universities and counties, with a higher magnitude in the case of universities and with a smaller magnitude across counties.

### 4.3. Discussion Based on the Results of the Estimated Models

In an increasingly knowledge-based globalized world, higher education, advanced training, and skill development are fundamental characteristics of the future. For developing students’ skills, universities deliver programs that better prepare them to straightforwardly respond to societal and labor market needs once they graduate by creating, refining, and adapting courses within their undergraduate and graduate programs [31]. To increase the efficacy of the education system, specialized skills need to be developed all throughout the workforce and this can be achieved by providing students with graduate education [32].

Thus, the decision of applying to a master’s program can be seen from two perspectives: on the one hand, the decision comes from the students’ desire to specialize in their specific field [31]; on the other hand, this decision comes from the desire to supplement their undergraduate degree by generalizing their knowledge with an advanced degree [31].

At the time of hiring, employers assess if a person has the potential for development, wherein the foremost critical aptitude considered when it comes to advancement is the capacity to acquire new skills [33] and for this precise reason, employers are more inclined to hire “demonstrated learners” who have a record of learning through formal instruction [32].

Students consider the importance of pursuing graduate education in relation to expectations from employers to recognize signals sent by graduate degrees and the expected earning premium associated with a graduate degree [32].

In our scientific demarche of investigating what encourages longer educational careers in tertiary education, the empirical results revealed three categories of determinants: individual characteristics, university characteristics, and county particularities.

**Figure 3:**
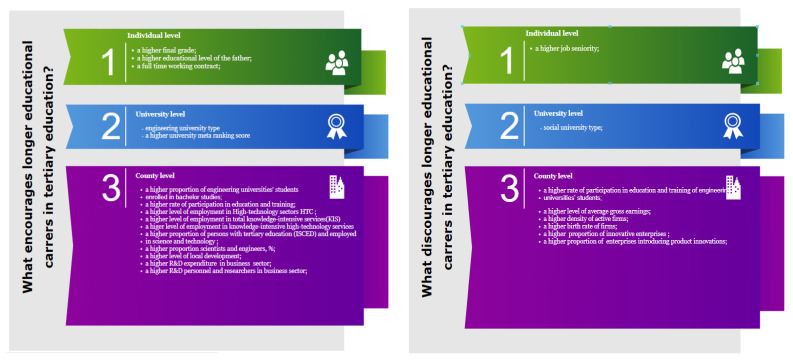
Summary of the main findings of our research.

Therefore, at the individual level, students with higher academic performances, with fathers with a high levels of education, with a working with full-time contract, and without a significant job seniority are more inclined to enroll in a future master’s program. These results are consistent with many other research findings [10,11,12,13,14,26,27], showing that previous academic performances and parental education as measures of the cultural capital of students influence educational decisions. Individual characteristics related to labor market participation that act as predictors for enrolment in master’s programs are consistent with the human capital theory. Pursuing a higher level of education is seen as a way for acquiring specialized skills valuable in the workplace, especially for those who have no working experience. A summary of our most relevant findings is provided in Figure 3.

As university determinants, previous studies showed that the satisfaction of students with educational experience shapes their predisposition to continue education and this can be highlighted by perceived performances and outcomes of educational institutions [17]. Our empirical evidence pointed out the positive effect of the university performance score on the decision to enroll in a master’s program. Another characteristic of influence is the type of university, with students from engineering universities being more inclined to enroll in a master’s program. The results subscribe also to other studies that highlight the influence of the context for further pursuing education, with a focus on the quality of education and on the important variations induced by the major on the predisposition to pursue a master’s degree. [14,15,16,17]. At the county level, three main categories of determinants have been revealed. In terms of educational indicators, we can point out the influence of the proportion of students from engineering universities enrolled in bachelor studies and the participation rate in education and training as the most relevant factors affecting the decision of applying to a master’s program. This reveals the importance of participation in learning activities, whether formal, non-formal, or informal, undertaken on an ongoing basis with the aim of improving knowledge, skills, and competences for personal, social, and/or professional reasons. During the course of a person’s working life, it is increasingly necessary to develop existing skills and to learn new skills that are relevant to a specific job or which provide opportunities for new career paths. The results are in line with the study of Jiménez and Salas-Velasco [12]. Thus, local contexts with high participation in education and training, especially in engineering, are those that provide higher incentives to bachelor students to enroll in master’s programs. Our results point to the fact that a higher level of skills at the local level acts as a catalysator for increasing the demand for master-level education among bachelor students.

A second category of determinants are related to the characteristics of the labor market for highly skilled workers that influence students’ decision of enrolment in higher education levels (Kallio, [25]). Among the most relevant factors determining the decision of enrolment into a master’s program, we can mention the level of employment in high-technology sectors (HTC), total-knowledge intensive sectors (KIS), and knowledge-intensive high-technology sectors (KIS_HTC); the proportion of persons with tertiary education employed in science and technology; the proportion of scientists and engineers; the level of local development; and R&D expenditure and the number of both personnel and researchers in the business sector. All of these determinants positively influence the decisions of students to enroll in a master’s program, capturing the formal creation of new knowledge within firms most likely in science-based sectors where most new knowledge is created in or near R&D laboratories. These results have been confirmed also by the findings of English and Umbach [2], Jiménez and Salas-Velasco [12], and Kallio [25].

The last category of determinants is related to the characteristics of the business sector that influence students’ decision of enrolment in higher education levels (Kallio, [25]). Thus, factors such as the average gross earnings, the density of active firms together with the birth rate of companies, and the proportion of innovative enterprises or those introducing product innovations tend to decrease the probability of continuing the educational career, attracting students to the labor market. The results found their confirmation in the studies of Jiménez and Salas-Velasco [12] as well as Kallio [25].

The birth of new enterprises is often seen as one of the key determinants of job creation and economic growth. Enterprise births are thought to increase the competitiveness of a country’s enterprise population by obliging them to become more efficient in view of newly emerging competition. As such, they stimulate innovation and facilitate the adoption of new technologies, while helping to increase the overall productivity within an economy. Therefore, an innovative business sector could offer alternative incentives to Romanian students who become less inclined to follow the courses of a master’s program.

What it is important to point out is the fact that average gross earnings and the dynamics of innovations in the business sector have been found as most likely to decrease the probability of continuing the educational career, but the marginal effect of these determinants depends on the university type. However, students from engineering universities with high earnings working in innovative enterprises or introducing product innovations are more inclined to enroll in a master’s program in the near future. In sum, our findings enrich the human capital theory with respect to enrolment in master’s programs, suggesting that students from more knowledge-intensive economic contexts anticipate higher returns from the further accumulation of highly specialized skills provided by master’s programs, while dynamic economic environments that are not very knowledge-oriented provide satisfactory employment opportunities and earnings for graduates of bachelor studies, discouraging them to further invest in acquiring master-level skills. Our study confirms the human capital theory by showing that individuals are more willing to invest in acquiring specialized skills in contexts of high demand of such skills and are thus able to return their investment.

Since the main limitation of the study refers to the inclusion of only five counties, future research studies will take into account a higher number of universities and counties, applying two essential methods of feature selection, namely chi-squared testand mutual information, both having as a main specificity the fact that output variables and input variables are categorical. The feature selection is the process of reducing or decreasing the number of input variables while expanding a prediction or classification model and it involves the evaluation of the relationship between the target feature and each input feature following statistics.

## 5. Conclusions

Students’ commitment and engagement in the educational process are shaped by a dense combination of factors, with effects on educational attainment and on the length of their educational careers. Decisions of prolonging education by enrolling in master’s degrees are beneficial for both individuals and societies as such programs provide higher levels of specialized skills [2]. Longer educational careers are favored by a mix of factors acting at the level of individual, university, or wider environment. The main aim of our research has been to identify which are the main factors at the level of counties, universities, and that of students, which determine the likelihood of master’s program enrolment in Romania.

The empirical results revealed that the academic performance, the father’s level of education, the type of working contract, and the job seniority are individual-level determinants influencing the decision of enrolment in a master’s program. At the university level, the type of university and the university performance score positively impact the students’ decision to enroll in a master’s program.

At the county level, the empirical evidence pointed out the significance of determinants such as the proportion of students enrolled in bachelor studies; participation rate in education and training; employment level in high-technology sectors (HTC), total-knowledge intensive sectors (KIS), and knowledge-intensive high-technology sectors (KIS_HTC); proportion of persons with tertiary education employed in science and technology; proportion of scientists and engineers; local development; R&D expenditure and both personnel and researchers in the business sector; average gross earnings; density of active firms; birth rate of companies; and proportion of innovative enterprises or those introducing product innovations on the decision to enroll in a master’s program. These findings show that the demand for master-level education is driven by knowledge-intensive economic contexts and environments characterized by the expansion of education and quality educational programs, while predisposition to further continue education is higher among highly academic performers and those coming from families with a higher stock of education. The results are relevant for Romanian universities that can improve the relevance of their master’s programs in relation to the needs of other students and to the characteristics of the wider economic and social environment in order to improve enrolment in their master’s programs. Potential future studies will aim to extend the research at the level of more countries, especially for assessing the factors shaping enrolment in master’s programs within various educational systems and economic settings.

## Figures and Tables

**Table 1:** The main indicators used in the research analysis.

Indicator	Code	Source	Period
The decision to apply to a master’s program in the academic year of 2020/2021 (dependent variable) was measured using the following question: “After completing your current bachellor studies, do you plan to apply for a master’s degree program in the following academic year?” This was coded as 1 for students who answered ‘yes’ to the question and 0 otherwise.	Appl_master	Educational survey	2019
**Level 1 indicators**			
**Student-related variables**			
Gender: a dummy variable with a value of 1 for women and 2 for men;	Gender	Educational survey	2019
Age: a numerical variable (mean-centered: 22.07)	Age	Educational survey	2019
Area of the usual residence: a dummy variable with a value of 1 for urban areas and 2 for rural areas.	Residence	Educational survey	2019
Graduation final grade of the previous school year: a numerical variable (mean-centered: 8.81)	Final_grade	Educational survey	2019
The highest level of education of the father: a categorical variable with a value of 1—low level (ISCED 0–2: without education, primary education, and lower secondary education); 2—medium level (ISCED 3–4: upper secondary education and post-secondary non-tertiary education); and 3—high level (ISCED 5–8: short-cycle tertiary education, bachelor’s or equivalent level, master’s or equivalent level, and doctoral or equivalent level).	Father_high_edu	Educational survey	2019
Subjective income was measured using the following question: “If you think about the total monthly income of your household, how you meet the needs?” This was coded as 1 for persons who answered ‘easy enough’ to the question and 2 for persons who answered ‘’with difficulty”.	Subj_inc	Educational survey	2019
Type of contract: a categorical variable with a value of 1—full-time, 2—part-time, and 3—not working for salary or other cash income.	Empl_type	Educational survey	2019
Seniority—working experince: a numerical variable (mean-centered: 0.918)	Seniority	Educational survey	2019
Full-time salary expected for a person who graduated with a master’s degree diploma: a numerical variable (mean-centered: RON 3815)	Master_salary	Educational survey	2019
Share of unemployed expected for graduates with a master’s degree: a numerical variable (mean-centered: 10.04%)	Unempl_master_degree	Educational survey	2019
**Level 2 indicators**			
**University-related variables**			
University type: a dummy variable with a value of 1—engineering or 2—social.	Univ_type	Educational survey	2019
University score: a numerical variable (mean-centered: 5.89)	Univ_score	University Metaranking: 2019 Ranking of Universities in Romania (Available online: https://ad-astra.ro/wp-content/uploads/2019/11/Metarankingul_Universitar_2019.pdf (accessed on 3 August 2021))	2019
Student number in the master’s programs (budget + fee): a numerical variable (mean-centered: 4582 persons)	Univ_students	The platform of the Integrated Educational Register, Universities Statistics (Available online: https://rei.gov.ro/statistici-universitati-13 (accessed on 3 August 2021))	2018–2019
Number of master’s programs: a numerical variable (mean-centered: 112)	Master_progr_nb	National Authority for Qualifications (Available online: http://site.anc.edu.ro/statistica/ (accessed on 3 August 2021))	2019
**Level 3 indicators (all level 3 indicators have been mean-centered)**			
***Labour maket indicators***			
ILO youth unemployment rate, 15–24 years, %	ILO_UR	Regional Labour Force, Tempo database, NIS	2018
Registered unemployed/100 employees	Unempl/employ	Territorial statistics database, Tempo database, NIS	2018
Average gross earnings, RON	Ag_g_earnings	Tempo database, NIS	2018
Vacancy rate for professionals, %	Vac_rate_prof	Regional Labour Force, Tempo database, NIS	2019
Vacancy rate for technicians and associate professionals, %	Vac_rate_tech_prof	Regional Labour Force, Tempo database, NIS	2019
Employment rate for ISCED 5–8, age 15–64 years, %	Tertiary_empl	Regional labour market statistics, Eurostat	2019
Employment in high-technology sectors HTC (high-technology manufacturing and knowledge-intensive high-technology services), % of total employment	Empl_HTC	Regional Science and Technology Statistics database, Eurostat	2018
Employment in total knowledge-intensive services (KIS), % of total employment	Empl_KIS	Regional Science and Technology Statistics database, Eurostat	2018
Employment in knowledge-intensive high-technology services (KIS_HTC), % of total employment	Empl_KIS_HTC	Regional Science and Technology Statistics database, Eurostat	2018
Persons with tertiary education (ISCED) and employed in science and technology, % of active population	Tert.Ed.ST_empl	Regional Science and Technology Statistics database, Eurostat	2018
Scientists and engineers, % of active population	SCT_ENG	Regional Science and Technology Statistics database, Eurostat	2018
Density of active firms, % of active firms by 1000 people in the population	Firm_density	Tempo database, NIS	2018
Birth rate: number of enterprise births in the reference period (t) divided by the number of enterprises active in t—%	Birth_rate	Regional Business Demography Statistics database, Eurostat	2017
Share of high growth enterprises measured in employment: number of high growth enterprises divided by the number of active enterprises with at least 10 employees—%	High_growth_enter	Regional Business Demography Statistics database, Eurostat	2017
Nominal GDP per capita, thousand RON/inhabitant	County level	Tempo database, NIS	2019
***Higher education indicators***			
Students enrolled in bachelor studies, % of total enrolled school-aged population	Stud_prop_bach	Tempo database, NIS	2018
Students enrolled in bachelor studies of full-time education, % of total students enrolled in bachelor studies	Stud_prop_bach_full_time	Tempo database, NIS	2018
Number of tertiary education degree graduates (bachelor)	Grad_Bach	Tempo database, NIS	2017
Number of tertiary education degree graduates (bachelor) of full-time education	Full_time_grad_bach	Tempo database, NIS	2017
Participation rate in education and training (last four weeks) representing share of the population aged 25–64 years that received formal or non-formal education or training (during the four weeks preceding the survey), %	E&T_part_rate	Regional Education Statistics database, Eurostat	2019
**Innovation indicators**			
***Investments***			
Expenditures for education financed from the local budget (RON/inhabitant)	Loc_budg_exp_ed	Territorial Statistics database, NIS	2018
Intramural R&D expenditure (GERD) in the higher education sector, % of GDP	R&D_exp_educ_s	Regional Science and Technology Statistics database, Eurostat	2017
Intramural R&D expenditure (GERD), the business enterprise sector, % of GDP	R&D exp_bus_s	Regional Science and Technology Statistics database, Eurostat	2017
Intramural R&D expenditure (GERD), the public sector, % of GDP	R&D exp_pub_s	Regional Science and Technology Statistics database, Eurostat	2017
Total R&D personnel and researchers, the business sector, as percentage of total employment—numerator in full-time equivalent (FTE), %	R&D personnel-bus_S	Regional Science and Technology Statistics database, Eurostat	2017
***Innovation activities***			
Innovative enterprises as a percentage of total enterprises	Innov_enterp	Tempo database, NIS	2016
Enterprises introducing product innovations as a percentage of total enterprises (only product innovators)	Product_innov	Tempo database, NIS	2016
Enterprises introducing process innovations as a percentage of total enterprises	Process_innov	Tempo database, NIS	2016
Enterprises introducing product and process innovations as a percentage of total enterprises	Product_Process-innov	Tempo database, NIS	2016

**Table 2:** The decision of applying to a master’s program among students from different universities and from different counties.

County	Brasov		Bucharest		Cluj		Dolj		Suceava	
University	Intention of Applying to a Master’s Program		Intention of Applying to a Master’s Program		Intention of Applying to a Master’s Program		Intention of Applying to a Master’s Program		Intention of Applying to a Master’s Program	
	No	Yes	No	Yes	No	Yes	No	Yes	No	Yes
Bucharest Academy of Economic Studies			65.71%	34.29%						
National University of Political Studies and Public Administration			58.33%	41.67%						
Babes Bolyai University					66.67%	33.33%				
University of Agronomic Sciences and Veterinary Medicine of Bucharest			0.00%	100%						
University of Bucharest			28.57%	71.43%						
University of Craiova							49%	51%		
Politehnica University			4.26%	95.74%						
Ştefan cel Mare University of Suceava									46%	54%
Technical University of Cluj-Napoca					46.43%	53.57%				
Transilvania University	43%	57%								
Total										

Source: own calculation.

**Table 3:** Multilevel mixed-effects logistic regression models (random intercept models) of the enrolment decision of Romanian students to a master’s program.

	Model I		Model II		Model III		Model IV		Model V		Model VI		Model VII		Model VIII	
	β	$exp\left(\beta \right)$	β	$exp\left(\beta \right)$	β	$exp\left(\beta \right)$	β	$exp\left(\beta \right)$	β	$exp\left(\beta \right)$	β	$exp\left(\beta \right)$	β	$exp\left(\beta \right)$	β	$exp\left(\beta \right)$
Individual-Level Variables																
Gender (women)																
Men	0.074	1.07	0.013	1.013	0.007	1.007	0.010	1.01	0.02	1.03	0.03	1.03	0.04	1.04	0.03	1.03
Age (mean-centered: 22.07)	−0.013	0.98	−0.045	0.95	−0.044	0.956	−0.045	0.95	−0.04	0.96	−0.03	0.97	−0.048	0.95	−0.06	0.94
Residence area (urban)																
Rural	−0.400	0.67	−0.392 *	0.675	−0.389 *	0.677	−0.392 *	0.67	−0.39	0.66	−0.374	0.69	−0.384	0.68	−0.36	0.69
Final grade (mean-centered: 8.81)	0.250 *	1.28	0.261 **	1.29	0.261 **	1.29	0.261 *	1.30	0.27 **	1.31	0.275 **	1.32	0.266 **	1.30	0.26 **	1.29
Father’s highest level of education (low)																
Medium	0.308	1.36	0.248	1.28	0.250	1.28	0.252	1.28	0.26	1.29	0.277	1.32	0.172	1.18	0.13	1.14
High	0.754 **	2.12	0.709 *	2.03	0.709 *	2.03	0.709 *	2.03	0.72 *	2.02	0.713 *	2.04	0.656 *	1.92	0.62 *	1.85
Subjective income (easy)																
Hard	0.203	1.22	0.204	1.22	0.198	1.22	0.202	1.22	0.21	1.23	0.210	1.22	0.206	1.22	0.20	1.22
Working contract type (not working)																
Full-time	1.073 ***	2.92	1.03 ***	2.80	1.02 ***	2.77	1.025 ***	2.78	1.04 ***	2.83	1.04 ***	2.83	1.11 ***	3.02	1.11 ***	3.04
Part-time	0.374	1.45	0.369	1.44	0.363	1.43	0.365	1.44	0.37	1.44	0.37	1.45	0.382	1.46	0.38	1.46
Seniority (mean-centered:0.91)	−0.244 **	0.78	−0.231 **	0.79	−0.229 **	0.79	−0.231 **	0.79	−0.23 **	0.79	−0.223 **	0.79	−0.225 **	0.79	−0.22 **	0.79
Master’s degree graduate full-time salary (mean-centered: 3815)	0.0001	1.00	0.00009	1.00	0.0001	1.00	0.0001	1.00	0.0001	1.00	0.0001	1.00	0.0001	1.00	0.00001	1.00
Master’s degree unemployed proportion (mean-centered:10.04)	0.006	1.00	0.00438	1.00	0.0046	1.00	0.0045	1.00	0.004	0.99	0.004	0.99	0.002	0.99	0.002	1.00
University-Level Variables																
University type (engineering)																
Social			−0.269 *	0.763	−0.274 *	0.76	−0.272 *	0.761	−0.29 *	0.74	−0.32 ***	0.72	−0.65 ***	0.52	−0.78 ***	0.46
University score (mean-centered: 5.89)			0.047 *	1.05					0.06 **	1.06	0.067 *	1.06	0.049 *	1.05	0.054 **	1.05
Student number in the master’s programs (mean-centered: 4582)					0.0001	1.00										
Number of master’s programs (mean-centered: 112)							0.003	1.00								
County-level Variables																
Students enrolled in bachelor studies, full-time education, % of total students enrolled in bachelor studies									−0.21 *	0.81						
Engineering univ.* Students enrolled in bachelor studies, %									0.03 *	1.02						
Participation rate in education and training											3.054 **	21.21				
Engineering univ * Participation rate in education and training											−0.268 *	0.88				
Average gross earnings													−0.0017 ***	0.99	-0.0022 ***	0.99
Engineering univ. * Average gross earnings													0.0073 **	1.00	0.0008 ***	1.00
Empl_htc													0.417 ***	1.51		
Empl_kis															0.155 ***	1.16
Constant	−0.165	0.85	0.455	2.06	0.698	2.01	0.733	1.72	0.64	1.91	0.563	1.75	0.981	2.66	1.44 ***	4.24
Observations	476		476		476		476		476		476		476		476	
No. Of groups	10		10		10		10		10		10		10		10	
No. Of counties	5		5		5		5		5		5		5		5	
Log likelihood	−300.89		−299.91		−300.05		−300.08		−299.91		−300.05		−300.08		−294.50	
Random-Effects Parameters																
County variance (cons)	0.23		0.19		0.18		0.17		0.17		0.16		0.26		0.15	
University variance (cons)	0.809		0.74		0.75		0.76		0.56		0.42		0.95		0.97	
Vpc at university level ^1^ (%)	19.77%		18.36%		18.56%		18.76%		14.54%		11.32%		22.40%		22.76%	
Vpc at county level (%)	6.51%		5.15%		5.18%		4.91%		4.91%		4.63%		7.32%		4.36%	
Lr test	30.66 ***		23.10 ***		22.94 ***		22.82 ***		13.95 ***		7.22 **		12.3 ***		11.7 ***	
	**Model IX**		**Model X**		**Model XI**		**Model XII**		**Model XIII**		**Model XIV**		**Model XV**		**Model XVI**	
	β	$exp\left(\beta \right)$	β	$exp\left(\beta \right)$	β	$exp\left(\beta \right)$	β	$exp\left(\beta \right)$	β	$exp\left(\beta \right)$	β	$exp\left(\beta \right)$	β	$exp\left(\beta \right)$	β	$exp\left(\beta \right)$
Individual-Level Variables																
Gender (women)																
Men	0.038	1.04	0.078	1.08	0.063	1.06	0.037	1.03	0.038	1.03	0.06	1.06	0.06	1.06	0.052	1.05
Age (mean-centered: 22.07)	−0.052	0.95	−0.048	0.95	−0.049	0.951	−0.051	0.95	−0.54	0.94	−0.03	0.97	−0.046	0.95	−0.046	0.95
Residence area (urban)																
Rural	−0.376	0.68	−0.367 *	0.692	−0.376 *	0.686	−0.373 *	0.68	−0.36	0.69	−0.407	0.66	−0.385	0.68	−0.39	0.68
Final grade (mean-centered: 8.81)	0.263 **	1.30	0.314 **	1.37	0.296 **	1.34	0.265 *	1.30	0.26 **	1.30	0.287 **	1.33	0.293 **	1.34	0.28 **	1.32
Father’s highest level of education (low)																
Medium	0.174	1.19	0.245	1.27	0.209	1.23	0.219	1.24	0.20	1.22	0.212	1.23	0.196	1.21	0.17	1.18
High	0.650 **	1.91	0.648 *	1.91	0.654 *	1.92	0.666 *	1.94	0.65 *	1.91	0.690 *	1.99	0.660 *	1.93	0.66 *	1.93
Subjective income (easy)																
Hard	0.203	1.22	0.231	1.26	0.219	1.24	0.203	1.22	0.20	1.22	0.220	1.25	0.218	1.24	0.21	1.23
Working contract type (not working)																
Full-time	1.01 ***	3.00	1.14 ***	1.27	1.15 ***	3.16	1.074 ***	2.92	1.08 ***	2.96	1.11 ***	3.03	1.15 ***	3.16	1.14 ***	3.13
Part-time	0.381	1.46	0.402	1.91	0.393	1.48	0.380	1.46	0.38	1.46	0.39	1.48	0.391	1.48	0.386	1.47
Seniority (mean-centered: 0.91)	−0.226 **	0.79	−0.235 **	0.79	−0.232 **	0.79	−0.231 **	0.79	−0.23 **	0.79	−0.23 **	0.79	−0.229 **	0.79	−0.22 **	0.80
Master’s degree graduate full-time salary (mean-centered: 3815)	0.0001	1.00	0.0001	1.00	0.0001	1.00	0.0001	1.00	0.0001	1.00	0.0001	1.00	0.0001	1.00	0.00001	1.00
Master’s degree unemployed proportion (mean-centered: 10.04)	0.003	1.00	0.0003	1.00	0.0014	1.00	0.003	1.00	0.003	1.00	0.002	0.99	0.001	0.99	0.002	1.00
University-Level Variables																
University type (engineering)																
Social	−0.65 ***	0.523	−0.737 *	0.478	−0.753 ***	0.47	−0.499 *	0.606	−0.582 **	0.56	−0.56 **	0.58	−0.75 ***	0.47	−0.75 ***	0.473
University score (mean-centered: 5.89)	0.056 *	1.05	0.053 **	1.05	0.055 **	1.05	0.062 *	1.06	0.067 *	1.06	0.029	1.03	0.051 ***	1.05	0.045 **	1.05
County-level Variables																
Average gross earnings	−0.0014 ***	0.998	−0.0020 **	0.99	−0.0021 ***	0.99	−0.0016 *	0.99	−0.00016 *	0.98	−0.004 **	0.99	−0.001 ***	0.99	−0.0013 ***	0.99
Engineering univ.* Average gross earnings	0.0007 **	1.00	0.0008 ***	1.00	0.00087 ***	1.00	0.00048	1.99	0.0006 *	1.00	0.0006 *	1.00	0.0008 ***	1.00	0.00089 ***	1.00
Empl_kis_htc	0.351 ***	1.42														
Scientists and engineers, %			0.60 ***	1.82												
Tert.ed.st_empl					0.229 ***	1.26										
Density of active firms							−0.120 *	0.88								
Firms’ birth rate									−0.848 ***	0.428						
Gdp per capita											0.135 **	1.14				
R&d exp_bus_s													4.85 ***	128.7		
R&d personnel-bus_s															4.72 ***	113.1
Constant	1.086	2.96	0.99	2.69	1.039	2.82	0.969	2.63	1.08	2.96	0.544	1.72	0.951	2.59	0.953	2.59
Observations	476		476		476		476		476		476		476		476	
No. Of groups	10		10		10		10		10		10		10		10	
No. Of counties	5		5		5		5		5		5		5		5	
Log likelihood	−294.50		−293.87		−293.38		−298.03		−297.10		−297.50		−293.58		−294.17	
Random-Effects Parameters																
County variance (cons)	0.21		0.19		0.18		0.17		0.19		0.17		0.25		0.23	
University variance (cons)	0.96		0.81		0.58		0.31		0.382		0.44		0.45		0.49	
Vpc at university level (%)	22.58%		19.75%		14.98%		8.61%		10.40%		11.79%		12.03%		12.96%	
Vpc at county level (%)	6%		5.45%		5.18%		4.91%		5.45%		4.91%		7.06%		6.53%	
Lr test	17.3 ***		23.10 ***		22.94 ***		9.09 ***		4.98 ***		7.80 **		12.3 ***		11.7 ***	

Note: All coefficients are compared to the benchmark category, as shown in brackets. *** *p* < 0.01, ** *p* < 0.05, and * *p* < 0.1. ^1^ Variance partition coefficient measures the proportion of the total residual variance that is due to between-group variation.

**Table 4:** Multilevel mixed-effects logistic regression models (random intercept models) of the enrolment decision of Romanian students to a master’s program.

	Model XVII		Model XVIII	
	β	$exp\left(\beta \right)$	β	$exp\left(\beta \right)$
Individual-Level Variables				
Gender (women)				
Men	0.054	1.05	0.047	1.05
Age (mean-centered: 22.07)	−0.04	0.96	−0.044	0.95
Residence area (urban)				
Rural	−0.389	0.68	−0.383 *	0.68
Final grade (mean-centered: 8.81)	0.247 **	1.33	0.281 **	1.32
Father’s highest level of education (low)				
Medium	0.247	1.28	0.243	1.27
High	0.684 **	1.98	0.679 *	1.97
Subjective income (easy)				
Hard	0.22	1.24	0.214	1.24
Working contract type (not working)				
Full-time	1.08 ***	2.96	1.08 ***	294
Part-time	0.39	1.48	0.386	1.47
Seniority (mean-centered: 0.91)	−0.23 **	0.79	−0.232 **	0.79
Master’s degree graduate full-time salary (mean-centered: 3815)	0.0001	1.00	0.0001	1.00
Master’s degree unemployed proportion (mean-centered: 10.04)	0.002	1.00	0.0003	1.00
University-Level Variables				
University type (engineering)				
Social	−0.48 ***	0.613	−0.472 *	0.62
University score (mean-centered: 5.89)	0.041 *	1.04	0.047 **	1.05
County-level Variables				
Average gross earnings	−0.002 ***	0.998	−0.003 **	0.99
Engineering univ. * Average gross earnings	0.0005 *	1.00	0.0005 *	1.00
Innovative enterp.	−0.114 *	0.89		
Only produs innovators			−0.86 *	0.42
Constant	0.68	1.98	0.78	2.19
Observations	476		476	
No. Of groups	10		10	
No. Of counties	5		5	
Log likelihood	−298.13		−298.31	
Random-Effects Parameters				
County variance (cons)	0.19		0.23	
University variance (cons)	0.47		0.53	
Vpc at university level (%)	12.5%		13.87%	
Vpc at county level (%)	5.45%		6.53	
Lr test	4.3 *		7.10 ***	

Note: All coefficients are compared to the benchmark category, as shown in brackets. *** *p* < 0.01, ** *p* < 0.05, and * *p* < 0.1.

## Data Availability

The survey dataset is available upon request. University statistics are available in Metarankingul Universitar-2019. Clasamentul Universităților din România, available on https://ad-astra.ro/wp-content/uploads/2019/11/Metarankingul_Universitar_2019.pdf (accessed on 10 August 2021). County and regional-level indicators are available on the Territorial Statistics and Tempo databases of the National Institute of Statistics (NIS) as well as on Eurostat databases.
